# Exploring the shared biomarkers between cardioembolic stroke and atrial fibrillation by WGCNA and machine learning

**DOI:** 10.3389/fcvm.2024.1375768

**Published:** 2024-08-29

**Authors:** Jingxin Zhang, Bingbing Zhang, Tengteng Li, Yibo Li, Qi Zhu, Xiting Wang, Tao Lu

**Affiliations:** ^1^ School of Life Sciences, Beijing University of Chinese Medicine, Beijing, China; ^2^ Chinese Medicine School, Beijing University of Chinese Medicine, Beijing, China

**Keywords:** cardioembolic stroke, atrial fibrillation, bioinformatics, weighted gene coexpression network analysis, hub gene, machine learning, Mendelian randomization analysis

## Abstract

**Background:**

Cardioembolic Stroke (CS) and Atrial Fibrillation (AF) are prevalent diseases that significantly impact the quality of life and impose considerable financial burdens on society. Despite increasing evidence of a significant association between the two diseases, their complex interactions remain inadequately understood. We conducted bioinformatics analysis and employed machine learning techniques to investigate potential shared biomarkers between CS and AF.

**Methods:**

We retrieved the CS and AF datasets from the Gene Expression Omnibus (GEO) database and applied Weighted Gene Co-Expression Network Analysis (WGCNA) to develop co-expression networks aimed at identifying pivotal modules. Next, we performed Gene Ontology (GO) and Kyoto Encyclopedia of Genes and Genomes (KEGG) pathway enrichment analysis on the shared genes within the modules related to CS and AF. The STRING database was used to build a protein-protein interaction (PPI) network, facilitating the discovery of hub genes within the network. Finally, several common used machine learning approaches were applied to construct the clinical predictive model of CS and AF. ROC curve analysis to evaluate the diagnostic value of the identified biomarkers for AF and CS.

**Results:**

Functional enrichment analysis indicated that pathways intrinsic to the immune response may be significantly involved in CS and AF. PPI network analysis identified a potential association of 4 key genes with both CS and AF, specifically PIK3R1, ITGAM, FOS, and TLR4.

**Conclusion:**

In our study, we utilized WGCNA, PPI network analysis, and machine learning to identify four hub genes significantly associated with CS and AF. Functional annotation outcomes revealed that inherent pathways related to the immune response connected to the recognized genes might could pave the way for further research on the etiological mechanisms and therapeutic targets for CS and AF.

## Graphical Abstract

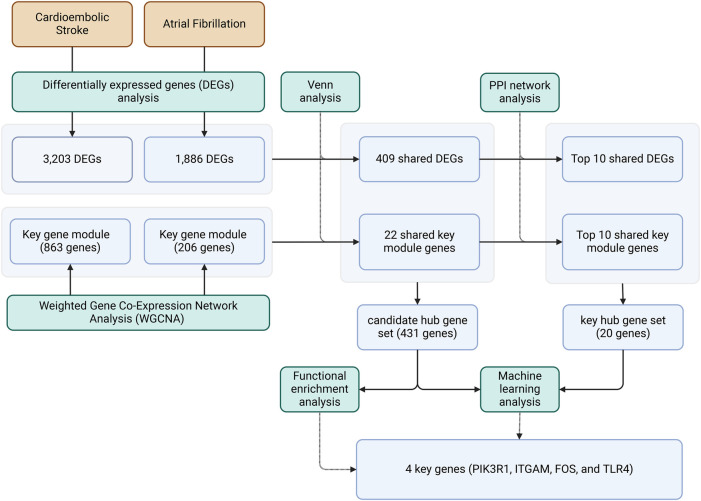

## 1 Introduction

Epidemiological evidence suggests that the incidence of ischaemic stroke in young adults (18-50 years) has increased substantially (1). There's an emerging agreement that numerous strokes with unidentified causes may not stem from cerebral disorders like microvascular emboli and hemorrhages, but rather from vascular emboli in other organs. The role of cardiac embolism in ischemic stroke is progressively increasing (2). Cardioembolic stroke (CS), which accounts for 20%–25%, is the most severe subtype of ischemic stroke. Characterized by a poor outcome and high recurrence, its primary causes include atherosclerosis, patent foramen ovale, and atrial fibrillation. Clinical studies using extended rhythm monitoring indicate that unexplained embolic strokes originate from Subclinical Atrial Fibrillation (SAF) (3), with Atrial Fibrillation (AF) increasing the risk of stroke by fivefold (4). One quarter of stroke cases involve Cardioembolic Stroke associated with Nonvalvular Atrial Fibrillation (NVAF) (5). Additional research indicates that Atrial Fibrillation (AF) is the predominant cause underlying CS, with estimates suggesting AF accounts for 15% of global stroke cases (6). Concurrently, the incidence and prevalence of AF are expected to increase in the forthcoming years, posing one of the most significant challenges in epidemiology and public health (7). However, the co-morbidity mechanisms between CS and AF remain unclear, necessitating an extensive comprehension of their association.

Immune mediators induce the infiltration of multiple inflammatory cells within the ischemic lesion, contributing to further cerebral ischemic injury. In CS, immune processes involve brain tissue and the entire organism and are linked to disability and mortality (8). This intensified inflammatory response also contributes to the prothrombotic state linked to AF (9). These findings suggest a possible mechanistic correlation between CS and AF. Although existing clinically relevant studies have shown a correlation between CS and AF, the genetic and molecular biological processes underlying these pathophysiologic mechanisms have not yet been fully elucidated.

Weighted gene co-expression network analysis (WGCNA) is a analytical method for characterizing molecular mechanisms and reconstructing gene co-expression networks through topological overlap (10). In the realm of medical research, machine learning techniques are frequently leveraged to discern latent biomarker predictors and therapeutic targets within both tumorous and non-tumorous pathologies. This application empowers physicians to forecast patient outcomes and assess their receptiveness to subsequent treatments (11). The Gene Expression Omnibus (GEO) database, encompasses a vast collection of experimentally sequenced high-throughput genomic data contributed by researchers (12). WGCNA and machine learning approaches can be utilized for the analysis of gene expression in high-dimensional datasets across multiple sample groups. These methods enable the categorization of highly similar genes into distinct modules, which can subsequently be analyzed to determine the correlation between key modules or hub genes within these modules and the clinical traits of patients (13), as well as to uncover various pathological processes and their associated characteristic genes (14).

In this study, utilizing differential analysis of gene sets and the WGCNA approach, co-expression networks were constructed to identify gene network modules associated with CS and AF. Subsequently, protein-protein interaction (PPI) networks and network-based node-mining algorithms were employed to analyze and identify hub genes within these networks based on the enrichment of shared gene pathways. Several commonly used machine learning algorithms were employed to assess the diagnostic value of the identified hub genes/biomarkers for AF and CS. As a result, the common pathogenesis of CS and AF was elucidated, and our study may provide novel insights for subsequent investigations into mechanisms and hub genes. These findings may provide potential new targets for the diagnosis and treatment of CS and AF, thereby contributing to improved stroke diagnosis and prevention in clinical practice.

## 2 Materials and methods

### 2.1 Data sources and preparation

Gene expression profiles of CS and AF were obtained from the GEO database (www.ncbi.nlm.nih.gov/geo). Inclusion criteria included: (1) AF human atrial tissue and CS blood samples present in the dataset; (2) The dataset contained samples from at least 15 patients; (3) The dataset was sourced from articles published between 2012 and 2022. Detailed information is available in Table 1.

**Table 1:** Brief description of CS and AF source dataset.

Disease	Data chip	Sample size		Data source	Year
		Normal control	Disease		
CS	GSE58294	23	69	Gene expression in peripheral immune cells following cardioembolic stroke is sexually dimorphic	2014
AF	GSE41177	6	32	Region-specific gene expression profiles in the left atria of patients with valvular atrial fibrillation	2013
AF	GSE79768	12	14	Differential left-to-right atria gene expression ratio in human sinus rhythm and atrial fibrillation: Implications for arrhythmogenesis and thrombogenesis	2016
AF	GSE115574	31	28	Molecular signatures of human chronic atrial fibrillation in primary mitral regurgitation	2021

Filtering removed negative or zero values that did not impact the overall biological analysis and were not suitable for logarithmic transformation, whereas weak signals lacked the strength to indicate significant gene expression differences. Normalization rendered each gene expression value as independent data, facilitating subsequent computational processes. The R software (version 4.0.5) and Bioconductor package were utilized to process the original expression data, construct the expression matrix, and correspond probes with their respective gene symbols. Following the download of the chip dataset, the probe identification numbers (IDs) were converted to gene symbols. If a gene corresponded to multiple probes, they were grouped, and the group with the highest mean was selected. Robust Multi-array Averaging (RMA) was employed for background correction and imputation of missing values. Subsequently, the Median Absolute Deviation (MAD) was applied to identify genes exhibiting high expression variability for subsequent analyses, and the pre-processed data were further analyzed using R software.

### 2.2 Screening the overlapping of differential expression genes

In this study, we identified and analyzed differentially expressed genes (DEGs) from comprehensive gene samples derived from CS and AF. DEGs were meticulously identified in both the control and disease groups. Subsequently, the results were visually represented through volcano plots generated using R software. The statistical significance threshold for identifying differentially expressed genes in these plots was set at *P* < 0.05. The overlapping DEGs were then identified by taking the intersection of the DEGs from the AF and CS groups.

### 2.3 Weighted gene co-expression network analysis

#### 2.3.1 Hierarchical cluster analysis

In this study, genes falling within the top 1,000 of MAD were included in the analysis. Recognizing the potential bias introduced by outlier samples in modular analysis, an appropriate threshold was established to identify and eliminate these outliers using hierarchical clustering. The clustering analysis was executed using the hclust function from the STATS package in R, enabling the categorization of samples into distinct functional modules denoted by different colors.

#### 2.3.2 Scale-free network construction and intensity matrix calculation

In accordance with the scale-free topology criterion, a biologically meaningful scale-free network was constructed using the Soft Threshold exponent (β), which was computed through the Soft Threshold algorithm in the WGCNA package. The pickSoftThreshold function was employed to select the value of β for which SFT.R.sq is around 0.9, ensuring a robust and biologically relevant network. The Pearson correlation matrix was calculated for all gene pairs.

#### 2.3.3 Co-expressed and key gene module construction

We employed the Topological Overlap Matrix (TOM) in conjunction with the dynamic tree-cutting algorithm to identify gene modules. Utilizing the connection strengths obtained earlier, we calculated the Topological Overlap (TO), enabling the measurement of gene pair connectivity. A hierarchical average chain clustering method based on TO was utilized to discern gene co-expression modules. This method not only gauges the connectivity between gene pairs but also facilitates the grouping of genes with similar expression patterns.

Upon determining the gene grouping modules, we calculated the eigenvector value (Module Eigengene, ME) for each module. We performed calculations to integrate the correlation between genes and traits (Gene Significance, GS) and the correlation between module eigenvectors and gene expression profiles (Module Membership, MM) with the gene significance of the module itself (Module Significance, MS). Candidate genes' significance *p*-values were computed using the *t*-test, where GS was defined as the logarithm (lg) of the mediated *p*-value (GS = lgP) for each gene. MS was the average GS of all participating genes in the module. The module associated with the disease was identified as the one with the highest MS value. We utilized the Pearson algorithm to analyze the correlation between modules and clinical trait associations. We selected modules with the highest correlation with clinical traits as the key modules, which were visualized in the trait gene network.

### 2.4 Protein-protein interaction (PPI) network construction

Protein-protein interaction (PPI) network were established by STRING database and Cytoscape software (3.10.1). The PPI network was constructed by selecting the ones with scores greater than 0.15. Then the PPI file was imported into Cytoscape, and the PPI network was analyzed and visualized using the “CytoHubba” plug-in of the software. The common genes in the network (the genes with the most interactions) were filtered by Maximum Neighborhood Component (MNC) algorithm, and the 10 genes with the highest scores were filtered according to the MNC algorithm and defined as top hub genes.

### 2.5 Functional enrichment analysis of hub genes for CS and AF

The Gene Ontology (GO) analysis of DEGs was conducted to elucidate the roles of gene products related to biological processes (BP), cellular components (CC), and molecular functions (MF). Additionally, the KEGG pathway database was utilized to identify and describe the functions of molecules and genes. We utilized the Metascape database for extensive bioinformatics analysis of genes and proteins. Metascape allows for comprehensive biofunctional annotation, enrichment analysis, protein interaction network analysis, and drug response profiling. The collection of hub genes related to CS and AF was entered into the search field of Metascape, designating “H. sapiens” as the organism. Entries displaying three or more significantly enriched genes and a *P*-value less than 0.01 were deemed significant.

### 2.6 Construction of machine learning models

We evaluated the significance of candidate hub gene sets and biomarker genes in the diagnosis of CS and AF. Meticulously, we constructed two commonly used machine learning models: Random Forests (RF) and Support Vector Machines (SVM). The construction and subsequent analysis of these models were facilitated by the Scikit-learn library, an open-source Python library widely recognized for its extensive collection of efficient tools for machine learning and statistical modeling.

To assess the predictive performance of our biomarker-based models, we implemented a rigorous evaluation process that involved generating Receiver Operating Characteristic (ROC) curves to provide a comprehensive overview of the models' classification abilities across various thresholds. Additionally, we computed the corresponding Area Under the Curve (AUC) values, a metric that offers a single scalar representation of the models' overall performance. By utilizing these advanced analytical techniques, we aimed to elucidate the potential clinical relevance of candidate gene and hub gene sets within the diagnostic landscape of CS and AF.

### 2.7 Mendelian randomization (MR) analysis

The dataset for AF was retrieved from https://csg.sph.umich.edu/willer/public/afib2018/, while the stroke data was sourced from https://www.finngen.fi/en. The instrumental variables were chosen based on specific criteria: (1) a genome-wide significance level (*P* < 5.0 × 10^−8^); (2) an r2 value under 0.001 within a clumping window spanning 10,000 kilobases; and (3) an F-statistic score exceeding 10.

Mendelian randomization (MR) analysis employed five distinct methods: MR Egger, Weighted median, Inverse variance weighted, Simple mode, and Weighted mode. Assessments of heterogeneity were executed utilizing MR-PRESSO. To examine pleiotropic effects, the MR Egger approach was implemented. TwoSampleMR package was applied in the above analysis.

## 3 Results

### 3.1 Construction and processing of CS and AF datasets

The dataset for CS was obtained from the GEO database, specifically the GSE58294 (15) (PMCID: PMC4103830) series, which included a total of 92 samples: 23 normal control samples and 69 samples from individuals diagnosed with cardiogenic embolic stroke. In the case of AF, we utilized three datasets: GSE41177 (16) (PMID: 23183193), GSE79768 (17) (PMID: 27494721), and GSE115574 (18) (PMCID: PMC8538404), encompassing a total of 123 samples. A concise description of the source data is provided in Table 1, while the detailed information can be retrieved from the GEO database.

### 3.2 Identification of DEGs and key module genes of CS

**Figure 1:**
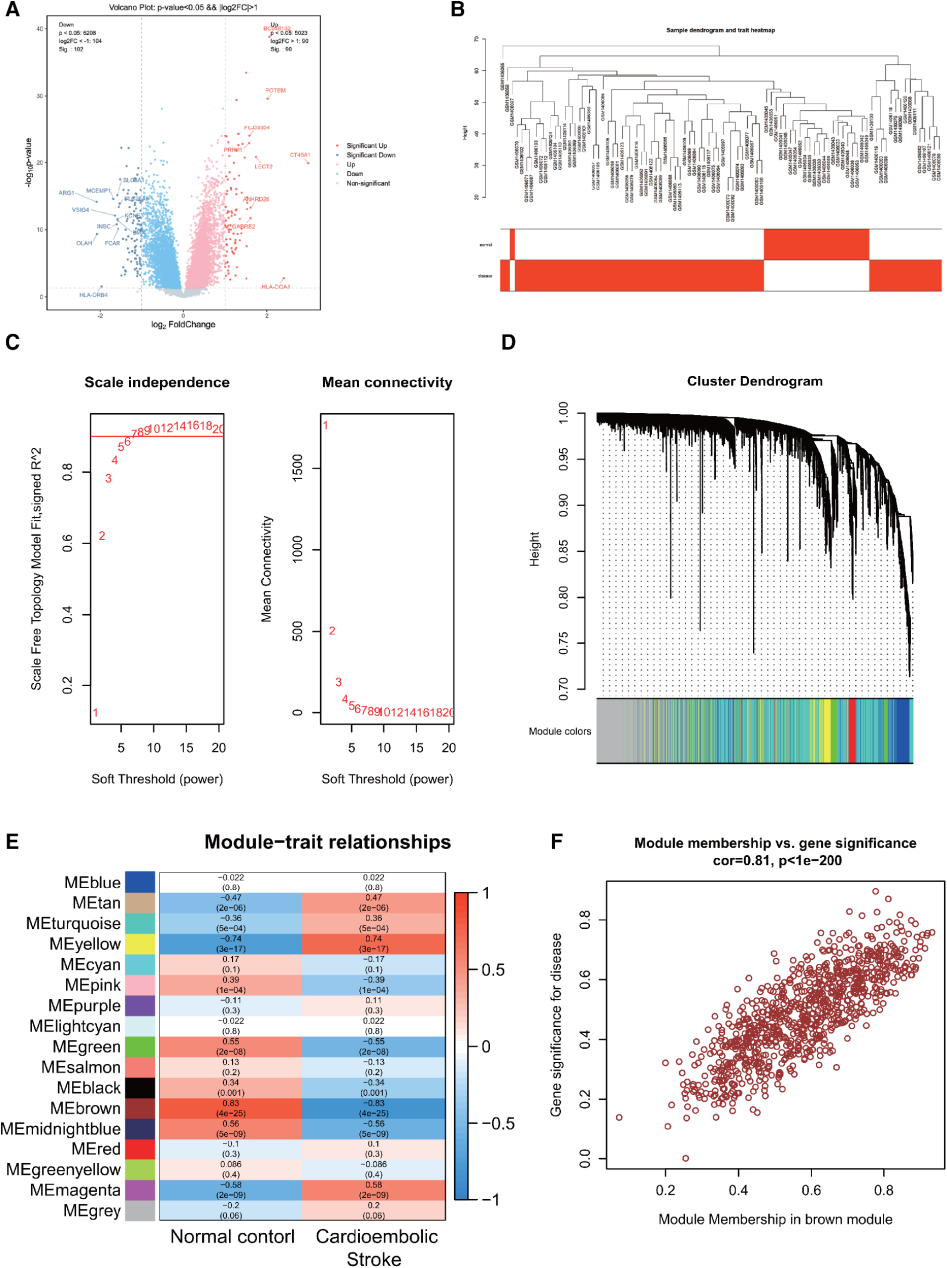
**(A)** Differentially expressed genes in CS. **(B)** Sample clustering dendrogram for CS. **(C)** Correlation between the fitting index and soft threshold (left), and the relationship between average connectivity and soft threshold (right). **(D)** Dendrogram of co-expression network module clustering in CS, with different colors indicating distinct modules. **(E)** Heatmap displaying the correlation between gene modules and clinical traits of CS; red signifies positive correlation, while blue indicates negative correlation. **(F)** Scatter plot of the brown module's module membership (MM) vs. gene significance (GS), with a correlation coefficient (cor) of 0.81, suggesting a strong association between the gene module and clinical features.

Using R software for data sorting and analysis of the CS dataset (GSE58294), we identified a total of 23,337 genes. After applying the criteria of |Log FC| > 0.4 and *P* < 0.05, we identified 3,203 differentially expressed genes (DEGs), with 1,667 being upregulated and 1,536 downregulated. Volcano plots depicting differential gene expression in CS (Figure 1A) were generated with a stringent |LogFC| threshold of 1. The analysis revealed the top 10 significantly differentially expressed genes (DEGs) associated with each condition. In the CS cohort, 9 genes exhibited notable upregulation, while 11 genes showed marked downregulation. The findings are summarized in Supplementary Table 1.

Following data preprocessing, the top 10,000 most variable genes in the CS dataset, as determined by the MAD score, were selected for analysis. A sample hierarchical clustering map was generated through WGCNA analysis, as shown in Figure 1B. The CS dataset samples were effectively classified into two clusters: normal (shown in white) and disease (shown in red). Notably, no outliers were detected. Figure 1C illustrates the relationship between the fitting index and the soft threshold (β). Specifically, a soft threshold (β) of 7 was identified as the first instance where the SFT.R.sq value exceeds 0.9, indicating an optimal fit (closer to 1). The second relationship depicted is between the average connectivity and β, confirming the biological relevance of the scale-free network properties in the CS. The soft threshold of *β *= 7 was identified as optimal for gene module delineation, resulting in the clustering of genes in the CS into 17 modules. The modules contained varying gene counts, ranging from 60 to 2,398, with the respective gene clustering tree depicted in Figure 1D.

The heatmaps were created to visually depict the correlation between gene modules and clinical features within the CS (Figure 1E), which shows that the brown module had the strongest correlation with CS (*r* = 0.83, *P* = 4e-25), with red indicating a positive correlation and blue indicating a negative correlation. The brown module associated with CS contained 863 genes. To pinpoint key genes associated with CS, the correlation between modules and clinical features was determined by calculating GS and MM. The x-axis represented the degree of association of genes within the module, whereas the y-axis depicted the degree of association of genes with the trait. The scatterplot of MM vs. GS in the brown module, revealing a correlation of 0.81 and *P* < 1e-200, demonstrated a high correlation between the gene module and clinical traits, as shown in Figure 1F.

### 3.3 Identification of DEGs and key module genes of AF

**Figure 2:**
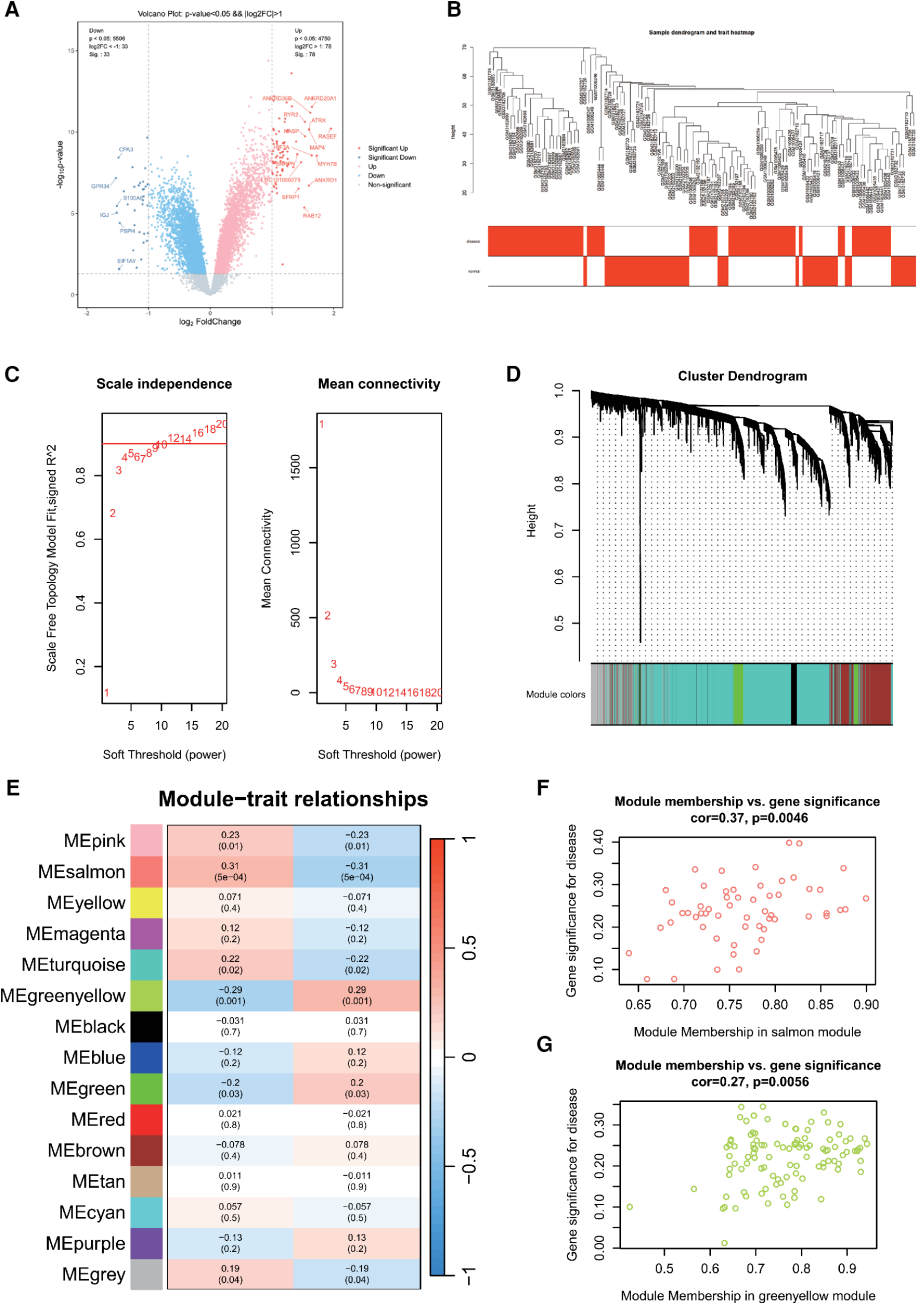
**(A)** Differentially expressed genes in AF. **(B)** Sample clustering dendrogram for AF. **(C)** Correlation between the fitting index and soft threshold (left), and the relationship between average connectivity and soft threshold (right). **(D)** Dendrogram of co-expression network module clustering in AF, with different colors indicating distinct modules. **(E)** Heatmap displaying the correlation between gene modules and clinical traits of AF; red signifies positive correlation, while blue indicates negative correlation. Scatter plot of the salmon **(F)** and greenyellow **(G)** module's module membership (MM) vs. gene significance (GS), with a correlation coefficient (cor) of 0.37 and 0.27, respectively.

We processed and analyzed three datasets: GSE79768, GSE115574, and GSE41177, which led to the identification of 23,335 genes employing a batch effect removal method. Employing the same filtering criteria, we discerned 1,886 DEGs, comprising 992 upregulated and 894 downregulated genes. Volcano plots depicting differential gene expression in AF (Figure 2A) were generated with a stringent |LogFC|threshold of 1. Within the AF cohort, 14 genes evidenced significant upregulation, contrasted by 6 genes displaying pronounced downregulation.

Next, 10,000 genes were identified in the AF dataset after preprocessing. The sample hierarchy clustering map was generated using WGCNA analysis, as shown in Figure 2B. Samples from the three AF datasets were categorized into two clusters: normal (represented in white) and disease (represented in red). Importantly, two outliers were detected. Using the scale-free topology criterion, the optimal soft threshold (β) was determined (Figure 2C). Specifically, when soft threshold (β) is set to 10, the first instance where SFT.R.sq exceeds 0.9 is observed.

Then, *β *= 10 was identified as the optimal soft threshold for gene module delineation. This led to the clustering of genes from the AF datasets into 12 modules. The gene counts within these modules ranged from 60 to 5,980, with the respective module cluster tree shown in Figure 2D. The heatmap was plotted to represent the correlation between gene modules and clinical features related to AF (Figure 2E). The top three modules were selected as key module, including salmon (*r* = 0.31, *P* = 5e-04), greenyellow (*r* = −0.29, *P* = 0.001) and pink (*r* = 0.23, *P* = 0.01). They contained 57, 104 and 145 genes, respectively. The scatterplot of MM vs. GS in the salmon and greenyellow module demonstrated a correlation between the gene module and clinical traits, as shown in Figure 2F.

### 3.4 Identification of shared hub genes between CS and AF

**Figure 3:**
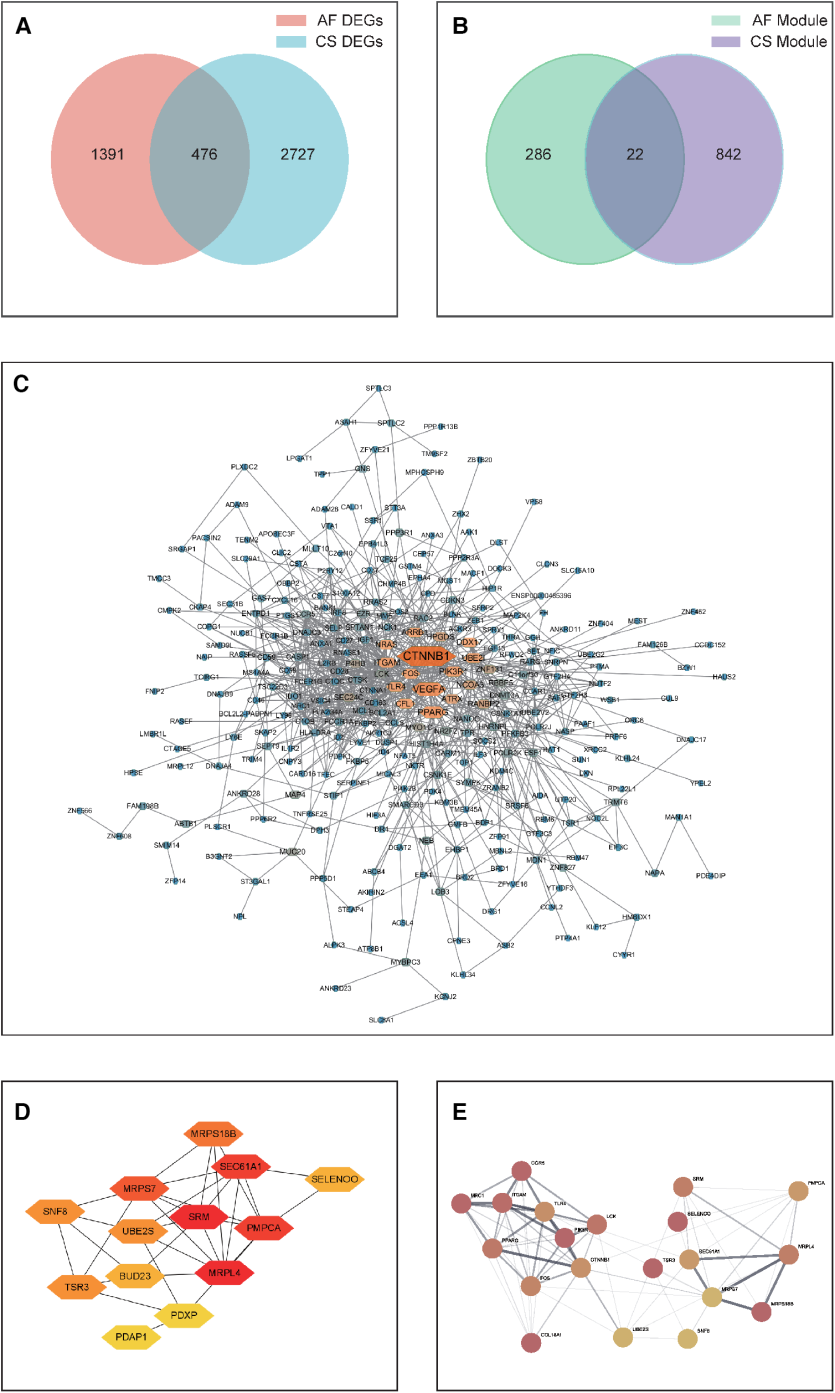
**(A)** A Venn diagram depicting the distribution of DEGs between CS and AF. **(B)** A Venn diagram showing the overlap of key module hub genes in CS and AF. **(C)** A PPI network graph of shared DEGs for CS and AF, with darker colors indicating higher interaction scores. **(D)** A PPI network graph of shared key module hub genes for CS and AF. **(E)** The top hub gene PPI network that integrates the top 10-ranking genes from the shared DEGs and module genes for CS and AF.

A Venn analysis was performed on all differentially expressed genes between the two diseases, as shown in Figure 3A. This revealed 476 overlapping genes. Eliminating duplicates and confirming gene IDs via Uniprot, an additional 67 genes were excluded, leading to a final count of 409 shared DEGs for AF and CS.

Subsequently, A PPI network was then constructed by visualizing the shared DEGs of CS and AF using the STRING database. Only interactions with experimentally validated combination scores greater than 0.15 were maintained. Visualized using Cytoscape software (Figure 3C), the resulting PPI network consists of 297 nodes symbolizing genes and 876 edges indicating gene interactions. The network topology was analyzed using the MNC algorithm in CytoHubba within Cytoscape software, assigning scores to each node based on their significance. This analysis led to the identification of the top 10 genes (CTNNB1, VEGFA, PPARG, ITGAM, PIK3R1, UBE2I, CFL1, TLR4, FOS, and ARRB1) as the principal shared DEGs. Biological information for these genes is presented in Supplementary Table 2.

Through WGCNA analysis, the brown module genes of CS and the three modules (salmon, greenyellow, and pink) genes of AS were recognized as the key module genes for CS and AF, respectively. We then conducted a Venn analysis between the two disease groups (Figure 3B). The analysis indicated that 22 genes were common as shared key module genes. Subsequently, a PPI network was constructed based on these genes, and consists of 13 nodes symbolizing genes and 31 edges, as depicted in Figure 3D. The MNC algorithm was also utilized on the network to pinpoint the top 10 shared module genes (SRM, MRPL4, PMPCA, SEC61A1, MRPS7, MRPS18B, UBE2S, SNF8, TSR3, SELENOO). Lastly, we merged the top 10 shared DEGs with top 10 shared module genes to form a PPI network (designated as the top-hub-gene network) using STRING, as presented in Figure 3E.

### 3.5 Enrichment analysis of shared hub gene between CS and AF

We aggregated the 434 DEGs and the 22 module genes to form a hub gene set, which altogether consists of 410 genes. In order to elucidate the potential molecular biological processes common to disease-related genes and, importantly, to uncover the co-morbidity mechanism between CS and AF, the functional analysis was performed using GO terms and KEGG pathway enrichment within the Metascape database. Table 2 displays the top 5 pathways associated with Cellular Component (CC), Molecular Function (MF), and Biological Process (BP) from the GO functional enrichment analysis, as well as the top 10 related pathways from the KEGG enrichment analysis.

**Table 2:** Pathway analysis of GO and KEGG enrichment for shared hub genes between CS and AF.

GO class	ID	Description	Count	*P*-value
BP	GO:0050867	Regulation of cell activation	29	3.98E-08
	GO:0002269	Leukocyte activation	27	6.46E-08
	GO:0002218	Innate immune response	31	9.55E-08
	GO:0002751	Endocytosis	23	1.32E-06
	GO:0002839	Positive regulation of immune response	25	1.91E-06
CC	GO:0072557	IPAF inflammasome complex	3	1.00E-05
	GO:0005925	Focal adhesion	18	2.40E-05
	GO:0009897	External side of plasma membrane	17	8.91E-05
	GO:0005911	Cell-cell junction	19	8.91E-05
	GO:0005769	Early endosome	17	9.12E-05
MF	GO:0019904	Protein domain specific binding	24	1.45E-05
	GO:0003841	Acyltransferase activity	25	2.45E-05
	GO:0003779	Actin binding	18	3.89E-05
	GO:0005159	Insulin-like growth factor receptor binding	4	5.50E-05
	GO:0001792	Immunoglobulin receptor activity	3	0.000199526
KEGG	hsa05133	Pertussis	9	1.23027E-06
	hsa04662	B cell receptor signaling pathway	8	2.51189E-05
	hsa05418	Fluid shear stress and atherosclerosis	10	2.95121E-05
	hsa05323	Rheumatoid arthritis	8	5.24807E-05
	hsa04613	Neutrophil extracellular trap formation	11	8.70964E-05
	hsa04613	T cell receptor signaling pathway	8	0.000114815
	hsa04141	Protein processing in endoplasmic reticulum	10	0.000165959
	hsa04670	Leukocyte transendothelial migration	8	0.000218776
	hsa04071	Sphingolipid signaling pathway	8	0.000323594
	hsa05202	Transcriptional misregulation in cancer	10	0.000436516

**Figure 4:**
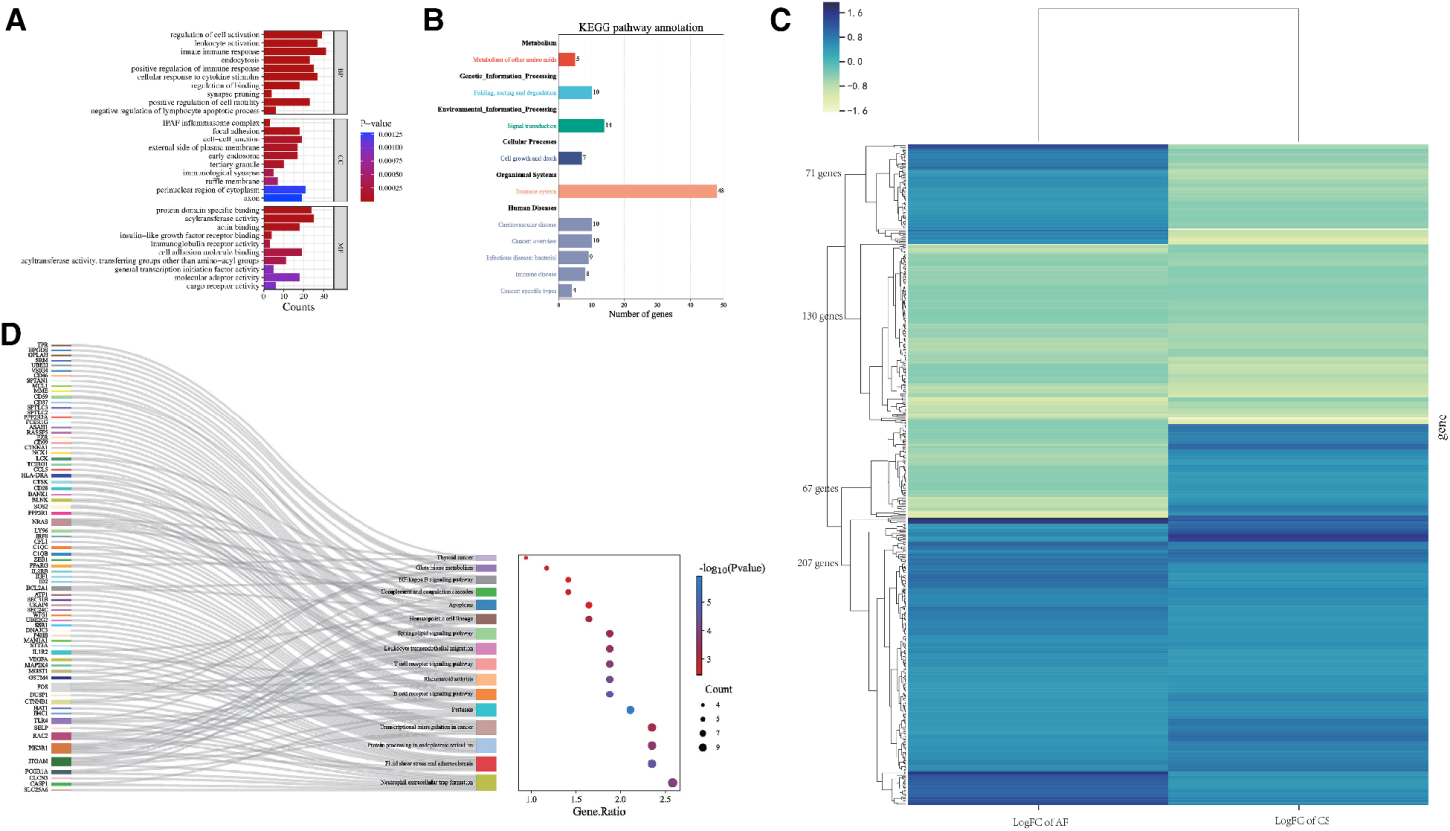
**(A)** Bar graph illustrating the GO enrichment analysis of the shared hub genes between CS and AF. **(B)** Categorization of KEGG pathways that are significantly enriched with the identified hub genes. **(C)** Heatmap of regulatory trends in differentially expressed overlapping genes between AF and CS. **(D)** Mulberry diagrams representing the KEGG pathway enrichment analysis for the hub genes, providing a visual representation of their involvement in various biological processes.

In terms of Biological Process (BP), the Gene Ontology (GO) terms were predominantly enriched in categories such as Innate Immune Response, Leukocyte Activation, and Regulation of Cell Activation. Regarding Cellular Component (CC), the genes were primarily enriched in the IPAF Inflammasome Complex and Cell-Cell Junction. For Molecular Function (MF), the genes showed enrichment in functions related to Protein Domain-Specific Binding and Acyltransferase Activity. The results were shown in Figure 4A.

The KEGG pathway enrichment analysis identified 64 pathways, which are summarized in a reclassification table in Figure 4B. We conducted a hierarchical heatmap analysis of overlapping gene expression in CS and AF, as shown in Figure 4C. Besides, the top 16 pathways visualized using a mulberry bubble map in Figure 4D. The analysis revealed a clustering of hub genes in several pathways, including Pertussis, B Cell Receptor Signaling, Fluid Shear Stress and Atherosclerosis, Rheumatoid Arthritis, and Neutrophil Extracellular Trap Formation. By integrating the outcomes from the top genes within the top-hub-gene network and the pathway classification depicted in the mulberry bubble map (Figures 3E, 4D), we identified the shared genes as potential biomarkers between CS and AF, which include PIK3R1, ITGAM, FOS, and TLR4. These genes were found to be enriched across multiple pathways, with a notable focus on the Neutrophil Extracellular Trap Formation pathway, which is essential for host immunity and plays a key role in metastatic diseases. The summary classification emphasized that the hub genes common to both CS and AF were primarily associated with inflammatory system-related pathways.

### 3.6 Assessing the diagnostic value of identified biomarkers for AF and CS

We integrated 409 shared DEGs for AF and CS with 22 shared key module genes to form a candidate hub gene collection that encompasses 431 genes. Concurrently, we constructed a key hub gene set composed of 20 genes by combining the top 10 key genes identified from each of the two PPI networks. To assess the diagnostic value of candidate hub genes and key hub genes identified from the PPI network in predicting AF and CS diseases, we developed models using Random Forest and Support Vector Machines. ROC curves were plotted, and model performance was evaluated based on AUC values, accuracy, precision, sensitivity, and specificity.

**Figure 5:**
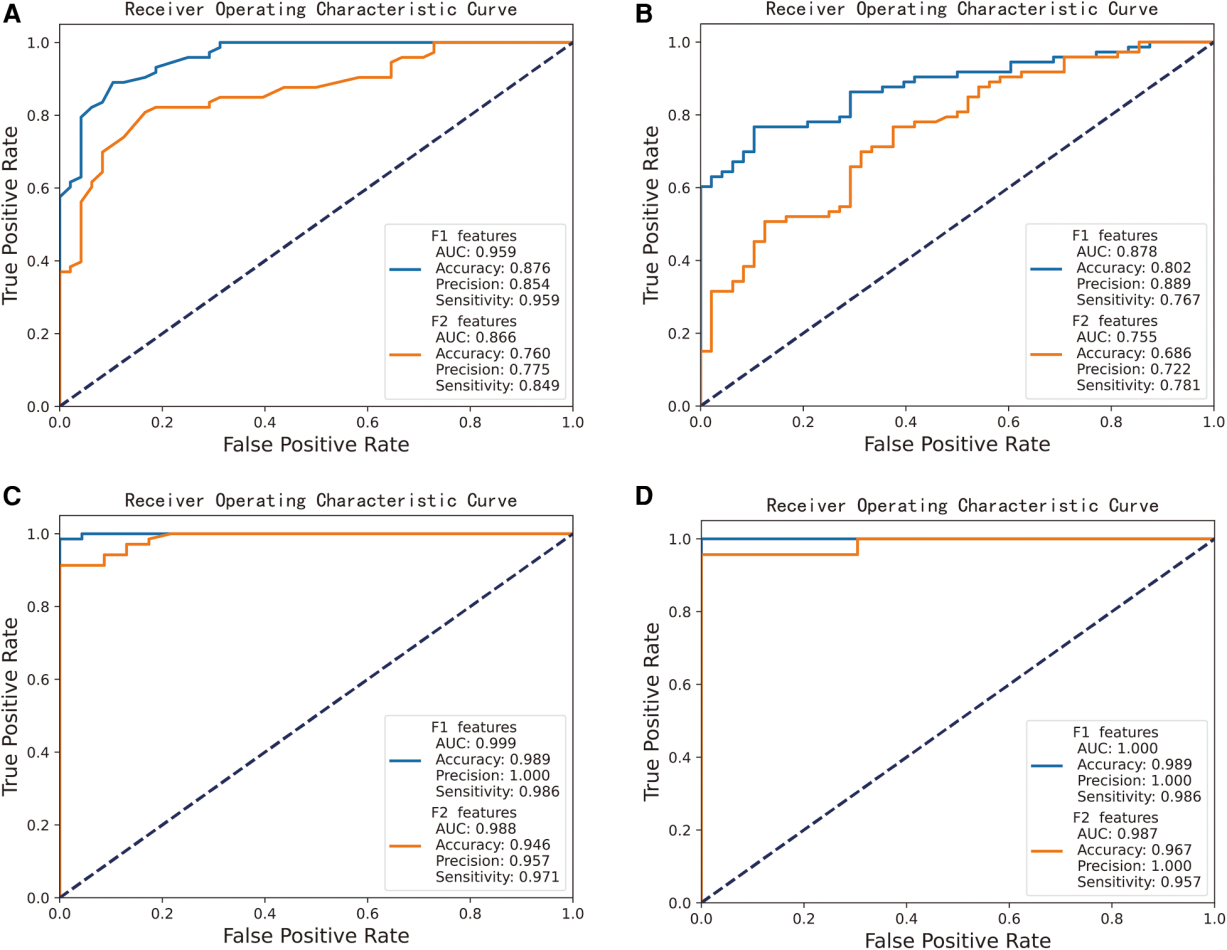
Performance of candidate gene-based machine learning models for predicting CS and AF. “F1 features” represents the potential hub gene, while “F2 features” signifies the crucial hub gene. **(A)** RF algorithm-based AF prediction model, yields AUC value of 0.959 (F1 features as input) and 0.866 (F2 features). **(B)** SVM algorithm-based AF prediction model, yields AUC value of 0.878 (F1) and 0.755 (F2). **(C)** RF algorithm-based CS prediction model, yields AUC value of 0.999 (F1) and 0.988 (F2). **(D)** SVM algorithm-based CS prediction model, yields AUC value of 1.000 (F1) and 0.987 (F2).

We randomly divided the samples from all groups and employed 5-fold cross-validation. The results indicate that both the candidate hub genes and the key hub genes achieved good predictive performance, as shown in Figure 5. The performance of the AF prediction model, built using the RF (Figure 5A) and SVM (Figure 5B) algorithm, yields an AUC value of 0.959 and 0.878 when the candidate hub genes are employed, and an AUC value of 0.866 and 0.755 when utilizing the key hub genes, respectively. The performance of the AF prediction model, built using the RF (Figure 5C) and SVM (Figure 5D) algorithm, yields an AUC value of 0.999 and 1.000 when the candidate hub genes are employed, and an AUC value of 0.988 and 0.987 when utilizing the key hub genes, respectively.

### 3.7 Causal estimates of AF on stroke based on MR analysis

**Figure 6:**
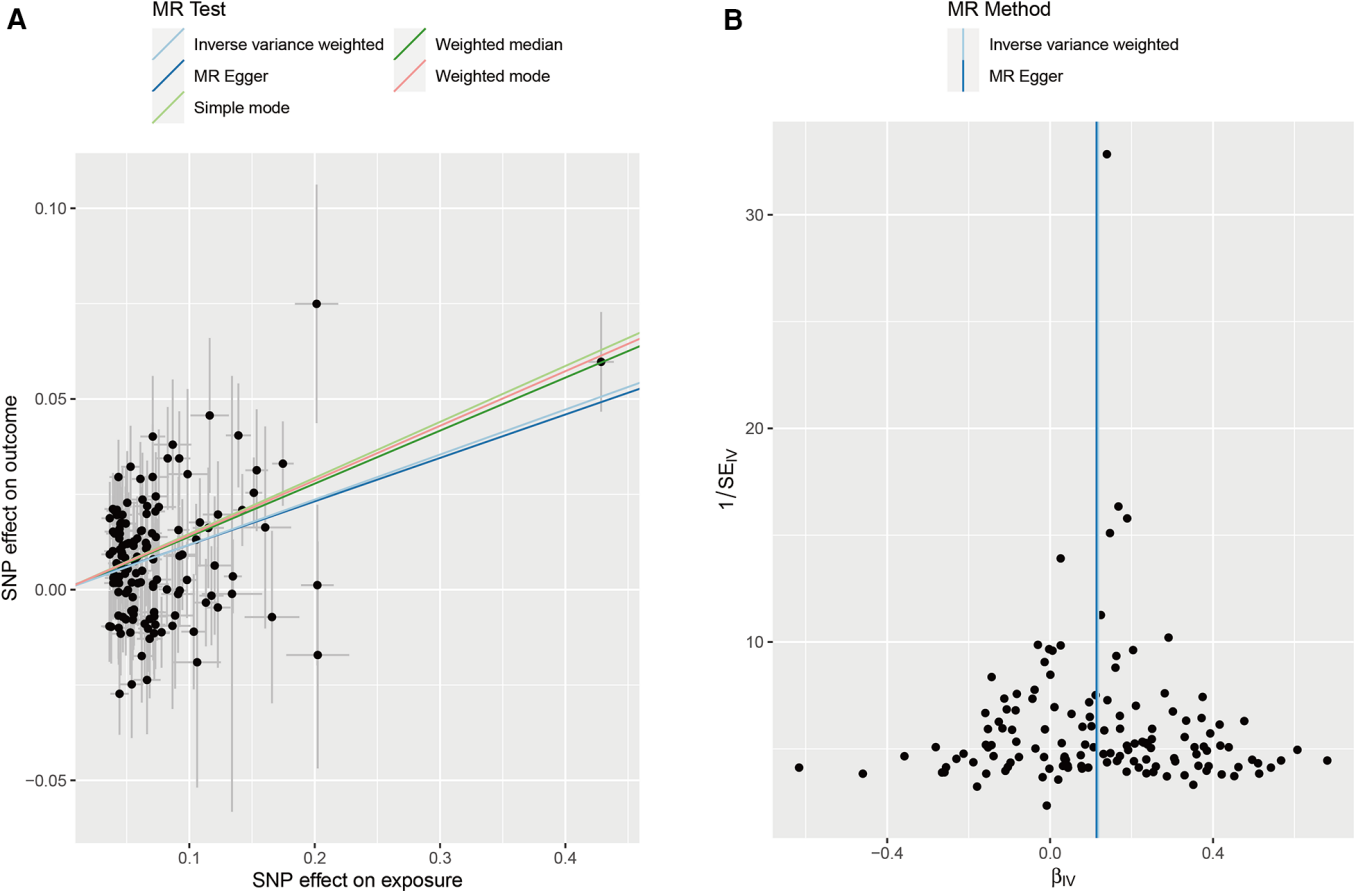
**(A)** Scatter plots of MR analyses. **(B)** Heterogeneity test of MR analysis.

We collected genome-wide association study (GWAS) summary data for AF from individuals (*N* = 60,620 AF cases and 970,216 controls). Additionally, we obtained GWAS data on stroke from individuals in the FINNGEN (*N* = 43,132 stroke cases and 297,867 controls). After screening, a total of 141 instrumental variables were obtained for AF, as shown in the Supplementary Table. Subsequently, five methods were used for MR analysis, with results presented in Table 3 and Figure 6A. The results indicate that AF has a causal association effect on stroke and can be considered a risk factor. Furthermore, using Inverse variance weighted and MR-Egger methods, heterogeneity was found in the exposure factor SNP (*P* < 0.05), as shown in Figure 6B. The MR-Egger intercept test was employed to detect the presence of pleiotropy. The results showed no evidence of pleiotropy (*p* = 0.8607).

**Table 3:** Causal estimates of AF on stroke.

MR methods	SE	OR (CI)	*P*-value
Inverse variance weighted	0.0144	1.1254 (1.0940, 1.1576)	<0.01
Weighted median	0.0243	1.1491 (1.0957, 1.2051)	<0.01
MR-egger	0.0281	1.1206 (1.0606, 1.1840)	<0.01
Simple mode	0.0567	1.1580 (1.0362, 1.2940)	<0.01
Weighted mode	0.0247	1.1540 (1.0995, 1.2112)	<0.01

In the subsequent phase, a reverse MR analysis was conducted, treating stroke as the exposure and AF as the outcome. This analysis aimed to investigate whether stroke increases the risk of AF. The results showed no causal association effect of stroke on AF. This finding is particularly interesting because it indicates a unidirectional relationship where AF influences stroke risk but stroke does not appear to increase the risk of AF. In conclusion, AF has a significant causal association effect on stroke.

## 4 Discussion

Stroke etiology is complex (19), defined by the abrupt occurrence of brain edema and neurological damage due to tissue injury in localized brain areas, leading to sympathetic activation, immunosuppression, as well as various complications (e.g., gastrointestinal hemorrhage, infections, and lower extremity deep vein thrombosis) and sequelae (e.g., cognitive and memory impairments, and limb mobility disorders). Clinically, strokes are classified into subtypes based on the “Trial of Org 10172 in acute stroke treatment” (TOAST) criteria, with CS being the most rapidly developing subtype, accounting for 30% of all strokes (20). Most patients with cryptogenic embolism are diagnosed with CS. CS etiology primarily involves heart diseases such as AF, heart failure, left ventricular embolism, mechanical aortic valve, infective endocarditis, aortic coarctation, and other cardiac disorders, with AF-related strokes making up over 79% of CS cases. Currently, primary treatments for cardiac stroke include anticoagulation, bridging therapy, intravenous thrombolysis, and mechanical thrombolysis, which have been shown to improve both early and long-term prognosis (21). Consequently, a multifaceted approach to studying stroke-related disease targets is necessary, paying attention to subtype-related diseases. For example, Malik et al. investigated genome-wide associations across multiple ancestries in 520,000 individuals, identifying 32 loci linked to stroke and its subtypes (22).

A typical bioinformatics algorithm for constructing gene co-expression networks from high-throughput gene expression microarray data is the WGCNA combined with machine learning techniques. Compared to conventional bioinformatics algorithms, WGCNA establishes the correlation between gene expression profiles and clinical information, thereby facilitating the exploration of novel therapeutic targets and offering new insights into the pathogenesis of comorbidities and combination therapy strategies. In a study by Huang K et al. (23), utilizing the WGCNA approach, it was discovered that STAT4, CX3CR1, COL1A2, and SH2D1B, with STAT4 and COL1A2 being significant mechanisms implicated in the co-morbidities of heart failure and depression, offer new targets for investigating the pathogenesis of heart failure and depression, as well as for treating these conditions. Wang et al. (24) identified the co-morbidity hub genes associated with systemic lupus erythematosus (SLE) and metabolic syndrome, and utilized the genes to construct a diagnostic model employing Random Forest and LASSO algorithms. Several studies have investigated the gene expression patterns related to clinical syndrome and stroke using WGCNA. In the work of Liu et al. (25), WGCNA was employed to identify OTULIN and NFIL3 as pivotal genes in heart failure-induced stroke. Zhao et al. (26) concluded from their WGCNA analysis that MAPK14 could act as a potential biomarker for CS and may have the capability to forecast the physiopathological condition of CS patients.

In our study, we initially retrieved clinical information and high-throughput gene expression data from the GEO database. We employed the Weighted WGCNA method to construct gene co-expression networks. Furthermore, we conducted differential gene analysis, PPI network analysis, and machine learning approaches to identify five hub genes significantly associated with CS and AF, including PIK3R1, ITGAM, FOS, CTNNB1, and TLR4. Further analysis showed that MRPL4, PMPCA, SEC61A1, MRPS7, MRPS18B, UBE2S, SNF8, and TSR3 are among the top 10 shared WGCNA genes.

For AF disease, we selected 3,257 significant module genes (greenyellow, turquoise, and salmon module) and 1,886 DEGs, resulting in an overlap of 998 genes. Further analysis revealed that VEGFA, UBE2I, and FOS are among the top 10 shared DEG hub genes. For CS disease, we selected 863 significant module genes (brown module) and 3,203 DEGs, resulting in an overlap of 414 genes. We conducted a hierarchical heatmap analysis of overlapping gene expression in CS and AF, as shown in Figure 4C. Our findings reveal that out of 475 differentially expressed genes, 130 are consistently down-regulated and 207 are up-regulated in both CS and AF. Furthermore, 71 genes are up-regulated in AF but down-regulated in CS, while 67 are down-regulated in AF and up-regulated in CS. Notably, 71% of these genes exhibit parallel expression trends in both conditions. With the exception of CTNNB1, all key regulatory genes identified align in their expression patterns. Consequently, CTNNB1 has been omitted from the hub gene list. Further examination of the four ultimately identified key shared genes was conducted to assess their differential expression patterns and WGCNA gene module. Notably, PIK3R1 and TLR4 satisfied these criteria.

The PPI network illustrates the complex interactions among proteins. Given the lack of direct overlapping genes between the two diseases, we sought to place these genes within a broader biomolecular interaction network to investigate their potential biological connections. This approach does not amalgamate these molecules *per se*, but rather capitalizes on extensive biological knowledge to observe the relationships exhibited by the two diseases within complex networks. As a result, we identified key genes (PIK3R1, ITGAM, FOS, and TLR4) using this methodology. We contend that this method surpasses the limitations of solely identifying key genes based on overlapping genes, which neglects the intricate biological relationships. This analytical technique supplements traditional Venn analysis, which only considers whether genes overlap, overlooking the complex interplay among them.

Subsequently, GO functional enrichment analysis revealed a significant correlation between each functional annotation process and immune and inflammation-related pathways. Additionally, KEGG pathway analysis indicated that hub genes associated with CS and AF were substantially enriched in immune response-related pathways. Notably, ITGAM were identified as common genes exhibiting genetic associations among various autoimmune diseases (27), consistent with the results from the GO functional enrichment analysis.

The above biological pathway findings are also consistent with several previous studies. Shi et al. (28) reported that TPA-mediated cerebral hemorrhage during IV thrombolysis for stroke, representing an immune invasion of the neurovascular unit, could be counteracted by precise immune modulation during therapy. In the study by Simone et al. (29), they analyzed the percentages of CD2 + T-bet + T cells and CD4 + GATA7 + T cells in the peripheral blood from patients with atherosclerotic thrombosis and CS, suggesting that circulating CD4 + T-bet + T cells might act as biomarkers for atherosclerotic thrombosis, indicating CS, and offering new perspectives on peripheral adaptive immune responses in acute stroke. Apart from the work by Ding et al. (30), who initially identified C1QC, VSIG4, and CFD as potential peripheral blood biomarkers for AF-related CS using bioinformatics approaches, no further studies exploring the molecular mechanisms underlying comorbidities associated with CS and AF were found.

Our study faces certain limitations: (1) The use of public databases with a modest sample size may introduce data bias; (2) The heavy reliance on bioinformatics algorithms for biomarker identification, without integrating findings from literature reviews and guidelines, is another constraining factor. Looking ahead, we aim to undertake large-scale clinical studies and perform animal model experiments to substantiate the proposed biomarkers. Furthermore, a systematic review will be conducted to distill common biomarkers, ensuring a multi-faceted evidence base for the disease under study.

In summary, our findings suggest that PIK3R1, ITGAM, FOS, and TLR4 are potential common biomarkers and therapeutic targets for both CS and AF. Further investigation into the immune response could elucidate the molecular mechanisms underlying these diseases, potentially offering novel insights for the management of their co-morbidities. However, future experimental validation is required.

## Data Availability

The datasets presented in this study can be found in online repositories. The names of the repository/repositories and accession number(s) can be found in the article/Supplementary Material.
