# Supplementary material for: Exploring the shared biomarkers between cardioembolic stroke and atrial fibrillation by WGCNA and machine learning
Source: Front Cardiovasc Med. 2024 Aug 29;11:1375768. doi: 10.3389/fcvm.2024.1375768 (PMC11390589; doi:10.3389/fcvm.2024.1375768)
Supplement: Supplementary file 1 [file Table1.doc]

Supplementary Table1 Differential gene expression information between CS and AF

| disease | Total number of genes | Number of genes screened | ↑DEGs gene number | ↓DEGs gene number | ↑significantly DEGs | ↑significantly  DEGs gene number | ↓significantly DEGs | ↓significantly DEGs gene number |
| --- | --- | --- | --- | --- | --- | --- | --- | --- |
| CS | 23337 | 3203 | 1667 | 1536 | PRR26,FLJ35934,GABRB2,ANKRD28,LECT2,POTEM,BC048132,HLA-DQA1,CT45A1 | 9 | VSIG4,INSC,SLC8A1,KCNE3,BMX,FCAR,SLC26A8,ARG1,OLAH,HLA-DRB4,MCEMP1 | 11 |
| AF | 23335 | 1886 | 992 | 894 | ANKRD36B,ATRX,MAP4,ANKRD1,NASP,RYR2,RAB12,LOC101060275,SFRP1,SNRPN,ANKRD20A1,RASEF,HIF3A,MYH7B | 14 | PSPH,EIF1AY,CPA3,S100A8,IGJ,GPR34 | 6 |

Supplementary Table2 Bioinformatics of key differentially expressed genes in CS and AF

| Symble | GeneCards Identifiers | Description | Related diseases |
| --- | --- | --- | --- |
| CTNNB1 | GC03P040551 | Catenin Beta-1 | Hairy stromal tumor  Colorectal cancer |
| VEGFA | GC06P043846 | Vascular Endothelial Growth Factor A | Diabetic Microvascular Complications Poe's syndrome |
| PPARG | GC03P012258 | Peroxisome Proliferator Activated Receptor Gamma | Familial deviant lipodystrophy III  Body mass index quantitative trait locus 11 |
| ITGAM | GC16P030449 | Integrin Subunit Alpha M | Systemic Lupus Erythematosus VI  Schwarzman phenomenon |
| PIK3R1 | GC05P067677 | Phosphoinositide-3-Kinase Regulatory Subunit 1 | Short syndrome and globulinemia VII  Autosomal recessive inheritance |
| UBE2I | GC16P001375 | Ubiquitin Conjugating Enzyme E2 I | Rett's syndrome  breast cancer |
| CFL1 | GC11M068156 | Cofilin 1 | Smith-Lemley-Opitz Syndrome  Muscular dystrophy |
| TLR4 | GC09P111571 | Toll Like Receptor 4 | Macular Degeneration  Aging-related X  Pertussis |
| FOS | GC14P073251 | Fos Proto-Oncogene/ AP-1 Transcription Factor Subunit | Congenital generalized lipodystrophy  osteoblastoma |
| ARRB1 | GC11M077303 | Arrestin Beta 1 | Placenta praevia  Nephrogenic syndrome with inappropriate antidiuresis |
